# Antiproliferative effect of methanolic extraction of tualang honey on human keloid fibroblasts

**DOI:** 10.1186/1472-6882-11-82

**Published:** 2011-09-26

**Authors:** Mohamad Shah Nurul Syazana, Ahmad Sukari Halim, Siew Hua Gan, Shaharum Shamsuddin

**Affiliations:** 1 Reconstructive Sciences Unit, School of Medical Sciences, Health Campus, Universiti Sains Malaysia, Kubang Kerian 16150, Kelantan, Malaysia; 2 Human Genome Centre, School of Medical Sciences, Health Campus, Universiti Sains Malaysia, Kubang Kerian 16150, Kelantan, Malaysia; 3 School of Health Sciences, Health Campus, Universiti Sains Malaysia, Kubang Kerian 16150, Kelantan, Malaysia

## Abstract

**Background:**

Keloid is a type of scar which extends beyond the boundaries of the original wound. It can spread to the surrounding skin by invasion. The use of Tualang honey is a possible approach for keloid treatment. The objective of this study was to determine the antiproliferative effect of methanolic extraction of Tualang honey to primary human keloid fibroblasts and to identify the volatile compounds in methanol extraction of Tualang honey.

**Methods:**

Crude Tualang honey was extracted with methanol and then dried using rota vapor to remove remaining methanol from honey. Normal and keloid fibroblasts were verified and treated with the extracted honey. Cell proliferation was tested with [3-(4,5-dimethylthiazol-2-yi)-5-(3-carboxymethoxyphenyl)-2-(4-sulfophenyl)-2H-tetrazolium, inner salt] (MTS) assay. Extraction of Tualang honey using methanol was carried out and the extracted samples were analysed using gas chromatography-mass spectrometry (GC-MS). The result was analysed using SPSS and tested with Kruskal-Wallis and Mann-Whitney tests.

**Results:**

Methanolic extraction of honey has positive anti proliferative effect on keloid fibroblasts in a dose-dependent manner. The presence of fatty acids such as palmitic acid, stearic acid, oleic acid, linoleic acid and octadecanoic acid may contribute to the anti-proliferative effect in keloid fibroblasts.

**Conclusions:**

The methanolic honey extraction has an antiproliferative effect on keloid fibroblasts and a range of volatile compounds has been identified from Tualang honey. The antiproliferative effect of keloid fibroblasts towards Tualang honey may involve cell signaling pathway. Identifying other volatile compounds from different organic solvents should be carried out in future.

## Background

Scar formation is the result of natural healing process which occurs after wounds, trauma, burns, surgical incision or disease. In order to close a wound, skin repair itself from normal to scar tissue and thus prevent infection [1]. Keloid is an end of the full spectrum of scar which extends beyond the boundaries of the original wound. It can spread to the surrounding skin by invasion. Clinical appearance of keloid is a raised growth and usually related with pruritus and pain. Keloid scars commonly occur only in humans. Keloid healing remains impaired since the pathogenesis of biochemical mechanisms of keloid is still unknown [2]. Development of keloid contains atypical fibroblasts and consists of overabundant of extracellular matrix components include collagen, fibronectin and certain proteoglycans.

Recently we reported the antimicrobial properties of Tualang honey [3], but the volatile compounds in Tualang honey from Malaysia and its effectiveness for treating keloid scar remain unknown. As Tualang honey is found in Malaysia's forest, various field of research on it has been widely carried out by researchers. The honey was used mainly to investigate its effect to certain diseases and compared this traditional treatment to modern treatment. Recently, Tualang honey has been used to determine its anticancer potential. For example, previous study has focused on the treatment of oral squamous cell carcinomas (OSCC) and human osteosarcoma (HOS) with various concentration of Tualang honey *in vitro*. Current modern treatment to these types of oral cancers such as surgery or radiotherapy can cause loss of function, disfigurement and reduced quality of life. From the finding, it is suggested that Tualang honey showed antiproliferative and apoptotic effect towards both oral cancer cell lines [4]. Human breast adenocarcinoma and cervical carcinoma (HeLa) cell lines were found potential to be treated with Tualang honey as it induced apoptosis of cancer cells via depolarization of the mitochondrial membrane. Thus, Tualang honey is found useful as an anticancer agent [5].

The Tualang tree which is also known as *Koompassia excelsa *found widely in Southeast Asia rainforests best known for the disk-shaped honeycombs which hang from its horizontal branches. It is mostly found in lowland forests of Peninsular Malaysia, southern Thailand, northern Sumatra and Borneo, and can grow to heights greater than 85 meters. The trees are valued by the locals due to its honey. In fact, a standing Tualang tree is more valuable for its honey than if it were felled for its timber. The honey from the combs of this tree is known as Tualang honey, and is produced by *Apis dorsata *or Asian rock bees [6].

Previously, continuous liquid-liquid extraction with diethyl ether has been used for the extraction of polar phenolic and acidic substances [7-9]. It has also been used for the determination of linalool derivatives in New Zealand honeys [10]. A simple liquid-liquid extraction method is advantageous as it does not involve heating which may lead to loss of volatiles or the formation of artifacts [11]. Therefore, extraction of honey samples using this method is more popular. The concentration of volatile constituents in honey are very low, therefore a sensitive technique such as gas chromatography-mass spectrometry (GC-MS) is needed. GC-MS is a simple instrument and requires very little time for quantification of compounds when compared to other methods such as high performance liquid chromatography. By using GC-MS methods, many important organic compounds have been detected in different types of honeys [12-16].

To our knowledge, no report has focused on determination of the volatile profile of Tualang honey. A continuous liquid-liquid extraction technique using five different organic solvents was selected due to its speed, low cost, and lack of sample heating.

## Methods

### Skin Sample Collection

Two types of samples which were primary normal human dermal fibroblasts (pNHDF) and primary keloid human dermal fibroblasts (pKHDF) were obtained from consented patients who underwent elective surgery at Hospital Universiti Sains Malaysia (HUSM). Sample collection was obtained after patients have agreed to give their skin for research purposes, have read and signed the informed consent forms. This study was approved by Research Ethical Committee Universiti Sains Malaysia (Human).

### Cell Culture

The skin samples were splitted into epidermal and dermal layers using dispase (2.4 units/mL DK-SFM) (GIBCO™, Invitrogen, USA) and incubated overnight at 4°C. The epidermal layer was further processed to obtain the keratinocytes and the dermal layer to obtain fibroblasts cell. The primary human epidermal keratinocytes were maintained in an Epidermal Keratinocyte Medium Defined (CELLNTEC, Switzerland) while the primary human dermal fibroblasts were maintained in Dulbecco's Minimal Eagle Medium (D-MEM) (GIBCO^®^, Invitrogen™, USA).

### Methanolic Extraction of Honey

Crude Tualang honey (50 g) was weighed and extracted with selected organic solvent, methanol. Pure methanol (100 mL) was poured to Tualang honey and the mixture was mixed well using shaker. Anhydrous disodium sulfate powder (Na_2_SO_4_, BDH Laboratory supplies, Poole, UK) was added (one third from the total honey/methanol mixture) to remove residual water. The sample then was saturated using filter paper which placed on filter funnel to remove other residual from the extracted honey. The extracts were then concentrated using rota-vapor (R-200, BUCHI) to remove methanol from honey. Concentrated honey was taken out from rota-vapor, kept on sterile glass tube and further dried using TECHNE Dri-Block to remove residual methanol. This extraction procedure was carried out based on the guidelines from National Poison Center, USM. The final concentration is 0.5 g/mL.

$$\begin{array}{ll} \text{Final concentration}:50\;\text{g} & -100\;\text{mL}\;\\ \text{X}\;\text{g} & -1\;\text{mL}\;\\ \text{X}\;\text{g} & =50\;\text{g}∕100\;\text{mL}\;\\ & =0.5\;\text{g}\;\\ & \; \end{array}$$

### Cell Treatment

Each primary normal human dermal fibroblast (pNHDF) and primary keloid human dermal fibroblast (pKHDF) cultures (100 μL) were seeded on separate 96-well plates and incubated for 24 hrs in a humidified incubator containing 5% CO_2 _incubator. After 70% confluence, cells were treated with different concentrations of extracted Tualang honey in each well. The concentrations were 12.5%, 6.25%, 3.13%, 1.56%, 0.78%, 0.39%, 0.20% and 0.10%. Positive control (Triton-X), negative control (untreated cells), background control (plain supplemented medium) and substance control (honey dilution) were included. The treatment was then incubated for 24, 48, and 72 hrs.

### Cell Proliferation Assay

Cell proliferation assay, MTS [3-(4,5-dimethylthiazol-2-yi)-5-(3-carboxymethoxyphenyl)-2-(4-sulfophenyl)-2H-tetrazolium, inner salt] (Promega, USA) is a colorimetric method for determining the number of viable cells in proliferation assays. MTS is bioreduced by cells into a formazan product that is soluble in tissue culture medium. MTS and phenazine methosulfate (PMS) solution were thawed. PMS solution (100 μL) was added to the 2 mL of MTS solution. The mixture was gently swirled to ensure complete mixing of the combined MTS/PMS solution. Combined MTS/PMS solution (20 μL) was pipetted into each well of the 96-well plates. The plates were incubated for 4 hrs at 37°C in a humidified 5% CO_2 _atmosphere. After 4 hrs, absorbance of each well was measured using ELISA reader with a test wavelength at 490 nm and a reference wavelength at 630 nm. The mean percentages of proliferated cells were calculated as below:

$$\begin{array}{c} \text{Percentage of proliferated cells by MTS}=\left(\text{O}{\text{D}}_{\text{sample}}∕\text{O}{\text{D}}_{\text{value}}\right)\times 100\;\%\\ \;\;\;\text{Where},\;\text{O}{\text{D}}_{\text{sample}}=\text{Optical Density of treatment samples}\\ \;\;\;\;\;\;\text{O}{\text{D}}_{\text{value}}=\text{Optical Density of control} \end{array}$$

### Analysis with GC-MS

Crude honey extract was added with 2 mL methanol (MEOH). The sample was vortexed for 3 min and centrifuged at 2500 rpm for 5 min. The clear top layer containing the honey methanolic extraction was transferred into a 1 mL autosampler vial before GC-MS injection. The extract was analyzed with GC-MS.

GC-MS analyses were performed on a HP6890 GC coupled with a HP5973 mass spectrometer. The column was a HP-5MS fused-silica capillary column (30 m × 0.25 mm i.d.; 0.25 μm film thickness), and helium running at a constant pressure of 14.5 psi was used as the carrier gas. One microliter volumes were injected using a splitless mode at an injector temperature of 250°C. The oven temperature was ramped from 35 to 280°C (1 min hold) at a rate of 25°C/min. The oven temperature was held at 310°C for 6 min following each analysis. The total run time for each sample was approximately 90 min. The GC-MS interface temperature was set to 280°C. Mass spectrometry mode was used during analytical scanning from 20-650 atomic mass unit (amu). The ion source temperature was set to 250°C. The blank was first injected, and was followed by the sample injection. The chromatograms obtained from the total ion count (TIC) were integrated without any correction for co-eluting peaks and the results were expressed as total abundance. All the peaks were identified based on mass spectral matching (≥ 90%) from both the National Institute of Standards and Technology (NIST) and Wiley libraries. Only compounds with 90% or greater spectral matching accuracy are reported.

### Statistical Analysis

The data from MTS assay was analyzed by SPSS (Statistical Packages for Social Science) version 18.0 for windows (Inc., Chicago, IL). The three independent experiments were analyzed using Kruskal-Wallis test while pairwise comparison was analyzed using Mann-Whitney test. Since the data was not normally distributed, non-parametric test was used. It was considered to be statistically significant if the p value < 0.05.

## Results and Discussion

### Proliferative Effect Determination of Tualang Honey Methanolic Extraction on pNHDF and pKHDF

**Figure 1:**
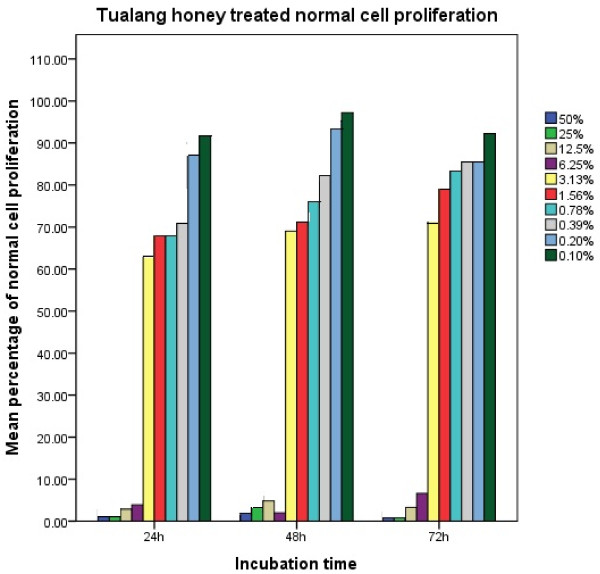
**Proliferation of normal cells following 24, 48 and 72 hrs of incubation with various concentrations of methanolic extract of Tualang honey**.

**Figure 2:**
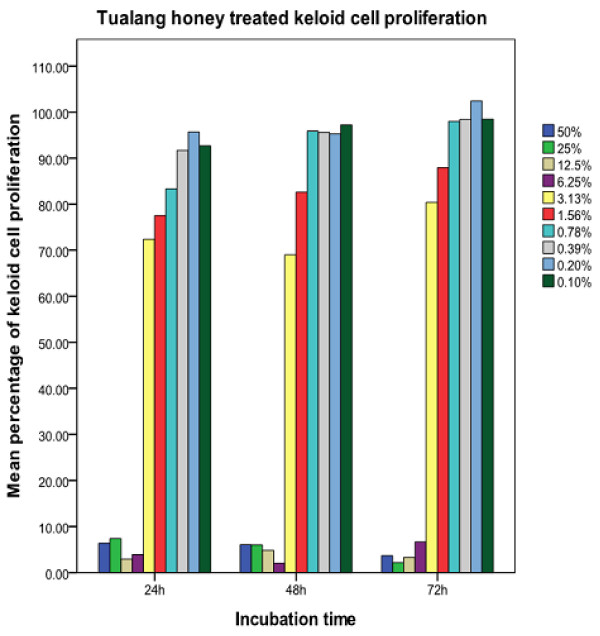
**Proliferation of keloid cells following 24, 48 and 72 hrs of incubation with various concentrations of methanolic extract of Tualang honey**.

Figures 1 and 2 indicated that the number of normal and keloid cell proliferation increases with the decrease in the concentration of Tualang honey. The decrease in proliferated cells was greater at higher concentration when compared to lower concentrations. The proliferative effect of pKHDF was similarly observed following 24, 48 and 72 hrs of exposure. Using Kruskal-Wallis test on ten concentrations of Tualang honey tested, only three concentrations (0.39%, 0.78% and 6.25%) showed significant difference, p < 0.05 following 24, 48 and 72 hrs of exposure to Tualang honey (Table 1) indicating that there were no significant differences in proliferative effect between the three time exposures.

**Table 1:** Cell proliferation in pKHDF after being treated with Tualang honey methanolic extracts at three different time of exposure

Concentration of Tualang honey (%)	Mean ± sd			p value
		
	24 h	48 h	72 h	
0.10	92.68 ± 7.21	97.19 ± 2.13	98.45 ± 2.80	0.99
0.20	95.71 ± 11.20	95.27 ± 3.00	102.40 ± 2.77	0.44
0.39	91.67 ± 10.51	95.62 ± 2.60	98.39 ± 2.10	0.05*
0.78	83.35 ± 11.72	95.84 ± 4.51	97.98 ± 3.89	0.03*
1.56	77.52 ± 9.98	82.61 ± 10.62	87.91 ± 2.65	0.47
3.13	72.40 ± 9.94	69.03 ± 20.08	80.36 ± 2.39	0.60
6.25	3.89 ± 2.61	2.00 ± 3.10	6.62 ± 7.32	0.02*
12.50	2.88 ± 3.08	4.81 ± 3.67	3.28 ± 1.29	0.39
25.00	7.34 ± 4.96	5.93 ± 4.12	2.11 ± 3.16	0.06
50.00	6.36 ± 5.65	6.00 ± 5.55	3.65 ± 3.10	0.37

Cells calculated based on control value

Data presented as mean ± sd; sd: standard deviation. Kruskal-Wallis test *Significant (p value < 0.05)

Table 2 showed the proliferative effect of treated pNHDF and pKHDF. From the result, it was found that there was a significant difference (p < 0.05) in cell proliferation of pNHDF and pKHDF at 0.10%, 1.56%, 6.25% and 12.50% of honey concentrations where the proliferative effects were significantly higher in pNHDF when compared to pKHDF. Meanwhile, the remaining concentrations showed no significant difference between their inhibition values. Reduced cell proliferation was found to be effective at 50%, 25%, 12.50% and 6.25% of concentration.

**Table 2:** Cell proliferation in pNHDF and pKHDF after being treated with Tualang honey between 0.10% to 50% concentration

Concentration of Tualang honey (%)	Mean ± sd		p value
		
	pNHDF	pKHDF	
0.10	98.23 ± 3.78	90.15 ± 5.86	0.00*
0.20	98.75 ± 3.99	91.29 ± 13.58	0.53
0.39	96.19 ± 7.64	91.31 ± 12.86	0.51
0.78	96.97 ± 7.14	90.26 ± 14.95	0.48
1.56	94.98 ± 7.31	83.08 ± 10.31	0.00*
3.13	68.13 ± 34.04	76.17 ± 12.42	0.54
6.25	0.00 ± 3.83	3.51 ± 6.99	0.00*
12.50	0.24 ± 0.33	0.68 ± 0.66	0.00*
25.00	3.55 ± 4.18	2.36 ± 11.61	0.94
50.00	6.46 ± 5.79	2.95 ± 5.14	0.30

Cells calculated based on control value

Data presented as mean ± sd; sd: standard deviation. ^a^Mann-Whitney test *Significant (p value < 0.05)

The antiproliferative effect of methanolic extract of honey at higher concentration suggests the possible effective concentration of methanolic honey extracts to treat pKHDF. Previous finding in which hepatocellular carcinoma cells were treated with chloroform/methanol extracts of honey found that the honey extracts exhibited antiproliferative effect on HepG2 cells. However with crude honey and petroleum ether honey extracts, there was increased proliferation of HepG2 cells [17]. In contrast, Ghashm *et al*. (2010) reported a different finding where they found that unfractionated honey induced cell-growth arrest of oral squamous cell carcinoma and human osteosarcoma blocked the cell cycle at the sub-G1 phase. These two different findings implicated that different cells types affect the effectiveness of either crude or extracted honey on cells.

Tualang honey acts on keloid fibroblasts by killing some of the fibroblast cells. This action caused reduction of the number of viable cells thus decreased the number of proliferated cells. Higher concentrations (50% and 12.5%) of Tualang honey was cytotoxic to fibroblasts cells but it became compatible to human fibroblasts following dilution of the honey and safe to treat human. Reduced rate of cell proliferation helps in reduction of cell growth and thus retard the cell growth. Cell growth retardation may minimize the size of keloid scarring. In previous finding, honey was proven to be a very effective agent for repressing the growth of bladder cancer cell lines *in vitro *[18]. The positive effect of Tualang honey to keloid fibroblasts suggest the role of Tualang honey in minimizing scarring.

### Determination of Compounds Detected from Honey Methanolic Extract

Several compounds were detected and identified from honey methanolic extracts using GC-MS. From the analysis, 27 compounds were identified, some of which may induce the anti-proliferative effects on keloid fibroblasts such as furfural alcohol, 4-oxo-5-methoxy-2-penten-5-olide, 3-Hydroxy-2-methyl-4H-pyran-4-one, 2-furoic acid, pyrocatechol, ketoisophorone, HMF, ethyl palmitate, oleic acid, linoleic acid and octadecanoic acid, 2-hydroxy-1-[hydroxymethyl] ethyl ester (Table 3).

**Table 3:** Twenty seven compounds detected with methanolic extracted honey

Detected compounds	Mean (Abundance)	SD (Abundance)	Coefficient of Variation (%)
furfural alcohol	1.15E + 08	44511402	38.59

Protoanemonine	2.75E + 07	725563.68	2.64

2-acetylfuran	2.27E + 07	5007827.8	22.10

2[5H]-furanone	2.40E + 07	1760998.5	7.34

2-hydroxycyclopent-2-en-1-one	1.18E + 08	12203793	10.37

4-oxo-5-methoxy-2-penten-5-olide	166949	86747.86	51.96

(5-methylfuran-2-yl)methanol	3948859	1153036.6	29.20

5-methyl furfural	2.27E + 07	2316696.8	10.21

2,4-dihydroxy-2,5-dimethyl-3[2H]-furan-3-one	2.59E + 07	7476341.8	28.88

Hyacinthin 1	1.58E + 07	619122.9	3.93

Hyacinthin 2	1.88E + 07	1412274	7.53

3-furoic acid methyl ester	2.49E + 07	381459.36	1.53

3-Hydroxy-2-methyl-4H-pyran-4-one	9458074	3279221.1	34.67

2-furoic acid	4.63E + 07	20121366	43.48

Ketoisophorone	1.90E + 07	24995107	131.75

3,5-dihydroxy-6-methyl-2,3-dihydropyran-4-one	3.07E + 08	31913403	10.39

5-acetoxymethyl-2-furaldehyde	2.27E + 07	964314.75	4.25

3,5-Dihydroxy-2-methyl-4H-pyran-4-one	2.93E + 07	6028182.9	20.56

Pyrocatechol	7686147	942361.21	12.26

HMF	1.75E + 09	439154881	25.04

palmitic acid	448760	38000.626	8.47

ethyl palmitate	126536	131167.6	103.66

5,5'-oxy-dimethylene-bis[2-furaldehyde]	2.87E + 07	3152723.3	10.98

linoleic acid	144296	9170.4678	6.36

oleic acid	105076	30963.499	29.47

Stearic acid	123760	15412.099	12.45

octadecanoic acid, 2-hydroxy-1-[hydroxymethyl] ethyl ester	43669	14338.711	32.83

Honey has been used not only for food but also as a traditional medicine. Recently, honey has gained public attention due to its potential ability in treating diseases and also in promoting health and well being [4]. Previous clinical studies have suggested that the positive effect of honey or sucrose may involve in minimizing keloids. Sucrose and mannose decreased in a dose-dependent-manner in both proα1(I) collagen and proα1(III) collagen mRNA levels [19,20]. Furan and pyran derivatives such as 2-acetylfuran, 5-methyl furfural, 3,5-dihydroxy-6-methyl-2,3-dihydropyran-4-one, (5-methylfuran-2-yl) methanol and 5,5'-oxy-dimethylene-bis[2-furaldehyde] which have been identified are the product of Maillard reaction which acts as an indicator of the absence of heat treatment and appropriate storage condition [21] and is not involved in the antiproliferative effect of honey.

Some types of honey depending on floral sources and geographical regions, may contain phenolic compounds. Phenolic compounds play a role as antioxidants, anti-carcinogenic, anti-inflammatory, anti-microbial, anti-atherogenic, anti-thrombotic and anti-proliferative potential [4,17,18]. However, phenolic compounds were not detected in methanolic extract of Tualang honey. Different methods need to be used to determine the phenolic content in honey for example using ethyl acetate extract and analyzing with thin layer chromatography (TLC) [22]. Thus, this could suggest that there are possible compounds from methanolic extract of honey that contribute to inhibition of cell proliferation. Tualang honey that has been extracted using methanol contains lipid including fatty acid esters such as oleic acid, linoleic acid and octadecanoic acid, 2-hydroxy-1-[hydroxymethyl] ethyl ester. It was previously reported that fatty acids show biological activity where they exhibit cytotoxicity against HeLa cells and retarded tumor growth [17]. This finding could support the presence of fatty acids in methanolic extract of honey that contribute to the inhibition of proliferative effect in keloid fibroblasts.

Palmitic acid can be toxic to cells and cause cell death in culture. Palmitic acid has been reported to inhibit CNS insulin signaling *in vitro *[23]. Palmitic acid could be one of the compounds involved in inhibiting keloid fibroblasts cell proliferation which was confirmed to be present in Tualang honey in our study.

This study is still at its elementary stage. The major problems are due to time constraint and the difficulties in maintaining primary cell culture. In future, collection of blood sample from keloid patients can be done instead of collection of keloid skin sample post surgery since blood sample is much easier to handle and large scale of sample is possible. As Tualang honey is proven to be non toxic and not harmful to be applied to the human body, it should be further investigated in order to determine what contributes to its unique properties for example its fatty acids compounds. In our study, since methanol extraction is found as the best solvent for honey extraction, this extraction has been used to treat fibroblasts. However, petroleum spirit, hexane or ethyl acetate can also be used as extracting solvent for the treatment on fibroblasts and their effectiveness can be compared with methanol extracts. This is helpful to confirm our first claim and strengthen the underlying results.

## Conclusion

As methanol extract was found to be the best organic solvent, methanolic extraction has been tested on fibroblasts cells for determining the ability of the honey extract to inhibit cell proliferation. Our findings showed the antiproliferative effect of methanolic honey extraction on keloid fibroblasts. Volatile compounds from methanol extract of honey has been identified using GC-MS and the presence of fatty acids from this extract such as oleic acid, linoleic acid, octadecanoic acid, palmitic acid and stearic acid may contribute to the antiproliferative effect in keloid fibroblasts. Further investigations in determining antiproliferative effect of keloid fibroblasts in relation to cell signaling pathway and identifying other volatile compounds from different organic solvents should be carried out. Investigations from molecular mechanism are also needed to determine the proliferative mechanism in order to obtain better findings. Even though honey is found to have the ability to reduce scar, scar recurrence may occur. We did not investigate if honey can prevent recurrence and this needs further investigation. In conclusion, the effort to treat keloid fibroblasts using traditional medicine such as honey should be pursued as keloid responds positively to Tualang honey treatment.

## List of Abbreviations

amu atomic mass unit, CO_2_: Carbon dioxide; °C: degree celcius; °C/min: degree celcius per minute; Na_2_SO_4_: Disodiumsulphate; ELISA: Enzyme-linked Imunosorbent Assay; ≥: equal to or more than; FAMA: Federal Agriculture Marketing Authority; GC-MS: Gas Chromatography-Mass Spectrometry; g gram: hrs hours; HUSM: Hospital Universiti Sains Malaysia; MEOH: Methanol; μL: microlitre; min: minute; mL: mililitre; MTS: 3-(4,5-dimethylthiazol-2-yi)-5-(3-carboxymethoxyphenyl)-2-(4-sulfophenyl)-2H-tertrazolium bromide, inner salt, nm nanometer, % percent: PMS: phenazine methosulfate; pNHDF: primary Normal Human Dermal Fibroblast; pKHDF: primary Keloid Human Dermal Fibroblast; rpm: revolution per minute; TIC: Total Ion Chromatogram.

## Competing interests

The authors declare that they have no competing interests.

## Authors' contributions

NSMS carried out the extraction process and runs the GCMS analyses as well as drafted the manuscript. SS and SHG participated in the design of the study and edited the manuscript. ASH participated in the acquisition of funding, design of the study, coordinating and monitoring of research. All authors have read and approved the final manuscript.

## Pre-publication history

The pre-publication history for this paper can be accessed here:

http://www.biomedcentral.com/1472-6882/11/82/prepub
